# Immune Cell Profiling Reveals a Common Pattern in Premetastatic Niche Formation Across Various Cancer Types

**DOI:** 10.1002/cam4.70557

**Published:** 2024-12-30

**Authors:** Shigeaki Sugiyama, Kanae Yumimoto, Keiichi I. Nakayama

**Affiliations:** ^1^ Department of Molecular and Cellular Biology Medical Institute of Bioregulation, Kyushu University Fukuoka Japan; ^2^ Department of Molecular Biology Graduate School of Medicine, Nagoya University Nagoya Japan; ^3^ Dynamic Chemical Life Science Laboratory, Department of Chemistry, Faculty of Science Kyushu University Fukuoka Japan; ^4^ Anticancer Strategies Laboratory, Advanced Research Initiative Institute of Science Tokyo Tokyo Japan

**Keywords:** diagnosis, machine learning, neoplasm metastasis, primary prevention, tumor microenvironment

## Abstract

**Background:**

Metastasis is the major cause of cancer‐related mortality. The premetastatic niche is a promising target for its prevention. However, the generality and cellular dynamics in premetastatic niche formation have remained unclear.

**Aims:**

This study aimed to elucidate the generality and cellular dynamics in premetastatic niche formation.

**Materials and Methods:**

We performed comprehensive flow cytometric analysis of lung and peripheral immune cells at three time points (early premetastatic, late premetastatic, and micrometastatic phases) for mice with subcutaneous implants of three types of cancer cells (breast cancer, lung cancer, or melanoma cells). The immuno‐cell profiles were then used to predict the metastatic phase by machine learning.

**Results:**

We found a common pattern of changes in both lung and peripheral immune cell profiles across the three cancer types, including a decrease in the proportion of eosinophils in the early premetastatic phase, an increase in that of regulatory T cells in the late premetastatic phase, and an increase in that of polymorphonuclear myeloid‐derived suppressor cells and a decrease in that of B cells in the micrometastatic phase. Machine learning using immune cell profiles could predict the metastatic phase with approximately 75% accuracy.

**Discussion:**

Validation of our findings in humans will require data on the presence or absence of micrometastases in patients and the accumulation of comprehensive and temporal information on immune cells. In addition, blood proteins, extracellular vesicles, DNA, RNA, or metabolites may be useful for more accurate prediction.

**Conclusion:**

The discovery of generalities in premetastatic niche formation allow prediction of metastatic phase and provide a basis for the development of methods for early detection and prevention of cancer metastasis in a cancer type‐independent manner.

## Graphical abstract

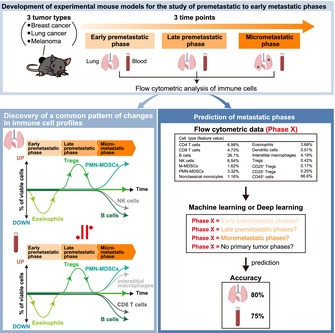

## 1 Introduction

About two‐thirds of deaths from solid tumors are due to cancer metastasis [1]. Prevention of metastasis is therefore a key approach to the elimination of such deaths. Metastatic cancer cells at sites of metastasis are surrounded by various immune‐suppressive cells such as tumor‐associated macrophages (TAMs), polymorphonuclear myeloid‐derived suppressor cells (PMN‐MDSCs), monocytic myeloid‐derived suppressor cells (M‐MDSCs), and regulatory T cells (Tregs). This microenvironment, which promotes metastatic tumor growth, is known as the metastatic niche [2]. TAMs, PMN‐MDSCs, and M‐MDSCs promote tumor development and metastasis by inhibiting T cells in a manner dependent on various cytokines and membrane‐associated molecules [3, 4, 5, 6]—including interleukin (IL)–10, transforming growth factor (TGF)–β, and CD40—that induce the differentiation of CD4 T cells into Tregs [7, 8]. These cells also produce arginase 1, which lowers the levels of L‐arginine, an amino acid essential for T cell responses [9, 10]. Furthermore, through the action of indoleamine 2,3‐dioxygenase, they metabolize tryptophan to kynurenine, which inhibits T cell proliferation [11, 12]. In addition, these cells express programmed cell death–ligand 1 (PD‐L1) and cytotoxic T lymphocyte‐associated antigen–4 (CTLA‐4), both of which inhibit T cells through cell–cell interaction [4, 6].

The premetastatic niche, defined as a supportive microenvironment already formed in distant organs before cancer metastasis, has attracted attention as a potential target for metastasis prevention [13, 14]. In mice transplanted with LLC (lung cancer) or B16 (melanoma) cell lines, both the expression of S100A8 and S100A9, a ligand of Toll‐like receptor 4 (TLR4), and the number of MDSCs were found to be increased in the lungs before metastasis. Administration of antibodies to S100A8/A9 attenuated such MDSC accumulation in the premetastatic lung, resulting in suppression of lung metastasis [15]. Prior treatment of mice with LLC cell‐derived exosomes resulted in a marked increase in the number of Tregs in the lungs, apparent before metastasis of transplanted LLC cells [16]. In addition, GW4869, an inhibitor of exosome generation and secretion, suppressed Treg differentiation and LLC cell metastasis in the lungs. The number of macrophages was found to be increased in the premetastatic lung of mice transplanted with the 4 T1 breast cancer cell line, and macrophage‐derived S100A4 induced fibroblast activation and deposition of extracellular matrix protein through activation of the ERK (extracellular signal–regulated kinase) signaling pathway [17]. Depletion of macrophages with clodronate liposomes inhibited such lung fibroblast activation and metastatic growth. These various studies thus implicate the premetastatic niche as a promising target for prevention of cancer metastasis. However, two key aspects of the premetastatic niche remain unclear: (i) Its generality. The described studies were performed with different cancer types and cell lines, but it remains unknown whether the cells that form the premetastatic niche differ among cancer types and cell lines. (ii) Its temporal progression. Although TAMs, M‐MDSCs, PMN‐MDSCs, and Tregs are known components of the premetastatic niche, the order in which these cells accumulate is uncharacterized.

We have now performed a comprehensive flow cytometric analysis at various time points for immune cells in the lungs and peripheral blood of mice transplanted with the breast cancer cell line E0771, the lung cancer cell line LLC or the melanoma cell line B16F10. We present three key findings: (i) The dynamics of immune cell composition show a common pattern during metastatic progression. (ii) The pattern of such changes in peripheral blood is similar to that in the lung. (iii) Each metastatic phase is characterized by a distinct immune cell profile independent of cancer type. On the basis of our findings and with the use of machine learning and deep learning, we predicted metastatic phase from immune cell profiles with approximately 75% accuracy. Our results provide the basis for the development of new approaches to the early detection and prevention of cancer metastasis across various cancer types.

## 2 Material and Methods

### 2.1 Mice

Experiments were performed with 7–16‐week‐old female mice on the C57BL/6J background, with the exception of those shown in Figure S4E, which were performed with 9–14‐week‐old male mice on the same background. C57BL/6J mice were obtained from The Jackson Laboratory. Generation of *CCR2* knockout mice was described previously [18].

### 2.2 Cell Culture

B16F10 and LLC cells (both provided by the Cell Resource Center of Tohoku University, Japan) and E0771 cells (CH3 BioSystems) were maintained at 37°C in RPMI 1640 medium (Wako) supplemented with 10% fetal bovine serum (NICHIREI), 1 mM sodium pyruvate (Gibco), penicillin (100 U/mL, Gibco), streptomycin (100 μg/mL, Gibco), 2 mM L‐glutamine (Gibco), nonessential amino acids (10 mL/L, Gibco), and 10 mM HEPES (Sigma‐Aldrich).

### 2.3 Tumor Cell Transplantation

Tumor cells (1 × 10^6^ LLC or B16F10 cells, or 2.5 × 10^5^ E0771 cells) were injected into the right back (LLC or B16F10) or the fourth mammary fat pad (E0771) of C57BL/6J mice.

### 2.4 Flow Cytometry

Flow cytometric analysis was performed as described previously [19]. A 25G winged needle was inserted into the right ventricle, and the lung was perfused with PBS (heparin 10 U/mL) until the lung was no longer bloody. Single‐cell suspensions were obtained from lung tissue by digestion for 60 min at 37°C with collagenase (1 mg/mL, Wako) followed by passage of the digested material through a 70‐μm cell strainer and lysis of red blood cells in the filtrate by treatment with 0.14 M NH_4_Cl in 0.01 M Tris–HCl (pH 7.5) for 5 min at room temperature. For sorting of peripheral blood cells, ~600 μL of peripheral blood was collected from the right ventricle into a tube containing 90 μL of 0.5 M EDTA, and red blood cells were lysed by treatment with 0.14 M NH_4_Cl in 0.01 M Tris–HCl (pH 7.5) for 30 min at room temperature. The Fc receptor of isolated cells was blocked by incubation for 15 min with antibodies to CD16/32 (2.4G2, BD Biosciences) before staining of the cells for 30 min with antibodies to CD45.2 (104, BioLegend), to CD11b (M1/70, BioLegend), to F4/80 (BM8, BioLegend), to Ly6G (1A8, BioLegend), to Ly6C (AL‐21, BD Biosciences), to CD11c (N418, BioLegend), to Siglec‐F (E50‐2440, BioLegend), to CX3CR1 (SA011F11, BioLegend), to CD3ε (145‐2C11, BD Biosciences), to CD4 (RM4‐5, BD Biosciences), to CD8α (53–6.7, BD Biosciences), to CD19 (6D5, BioLegend), to NK1.1 (PK136, BD Biosciences), to CD25 (PC61, BD Biosciences), to CCR2 (SA203G11, BioLegend), to TIGIT (1G9, BD Biosciences), to LAG‐3 (C9B7W, BioLegend), to CD73 (TY/11.8, BioLegend), to CTLA‐4 (UC10‐4B9, BioLegend), and to PD‐1 (29F.1A12, BioLegend). For intracellular staining, cells were treated with fixation and permeabilization solutions (Invitrogen) and stained with antibodies to Foxp3 (FJK‐16 s, Invitrogen) and to TGF‐β (TW7‐16B4, BioLegend). The stained cells were treated with 7‐aminoactinomycin D (7‐AAD, BD Biosciences) to exclude dead cells and were then analyzed with a FACSVerse, FACSAria, or FACSymphony A1 flow cytometer (Becton Dickinson).

### 2.5 ELISAs

Cytokine levels were quantified with a Mouse IL‐5 ELISA Kit (KE10018), Mouse CCL17/TARC ELISA Kit (KE10096), Mouse CCL22 ELISA Kit (KE10053), and Mouse MCP‐1 ELISA Kit (KE10006), all of which were obtained from Proteintech. The Mouse IL‐5 ELISA Kit was used to quantify serum IL‐5, and the Mouse CCL17/TARC ELISA Kit, Mouse CCL22 ELISA Kit, and Mouse MCP‐1 ELISA Kit were used to quantify CCL17, CCL22, and CCL2 in serum and lung.

### 2.6 UMAP

Normalized flow cytometry data (% of viable cells) were used for UMAP analysis with the UMAP function (n_components = 2, n_neighbors = 15, min_dist = 0.1, metric = ‘euclidean’) of umap‐learn library.

### 2.7 Machine Learning

Three machine learning (ML) classifiers (logistic regression, random forest, and support vector machine) were adopted to predict metastatic phase. Normalized flow cytometry data (% of viable cells) were cross‐validated with each ML classifier (number of splits was 5 and data were shuffled), and a mean accuracy score was calculated.

### 2.8 Deep Learning

Deep learning was performed with the use of the Keras library. Normalized flow cytometry data were split (training data: testing data = 4:1), and 20% of the training data were used as validation data for hyperparameter tuning. The model was constructed with the use of the Sequential() function, with two hidden layers (units of 10 and 5, respectively; the activation function was the ReLu function). The activation function for the output layer was softmax, and a dropout layer was placed before the output layer. Categorical_crossentropy was used for the loss function, Adam for the optimization algorithm, and categorical_accuracy for the evaluation function. An EarlyStopping (patience = 10, verbose = 0) function was applied to train the model (epochs = 500, batch_size = 30) so as to prevent overtraining.

### 2.9 Statistical Analysis

All statistical analyses were performed with EZR (Saitama Medical Center, Jichi Medical University, Saitama, Japan), which is a graphical user interface for R (The R Foundation for Statistical Computing, Vienna, Austria) [20]. More precisely, it is a modified version of R commander designed to add statistical functions frequently used in biostatistics. Quantitative data were compared between two or among three or more groups with the unpaired two‐sided Student's *t* test or Tukey–Kramer test, respectively. EZR software was used for correlation analysis. A *p* value of < 0.05 was considered statistically significant.

## 3 Results

### 3.1 A Common Pattern of Changes in Lung Immune Cell Composition During Metastasis

**FIGURE 1:**
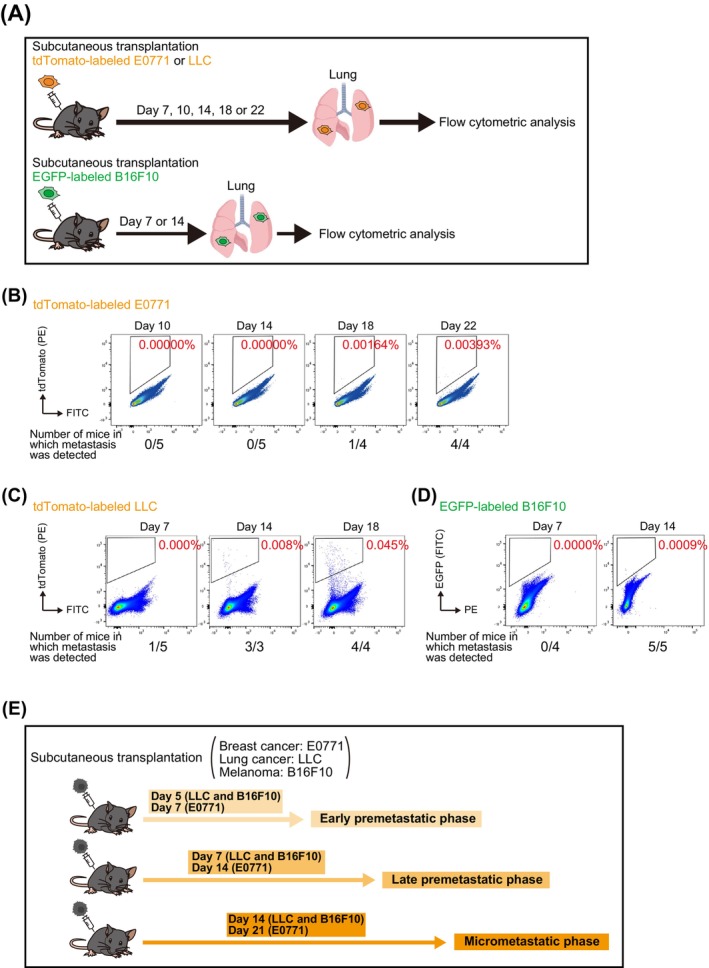
Development of experimental mouse models for the study of premetastatic to early metastatic phases. (A) Schematic representation of flow cytometric analysis to determine the timing of spontaneous metastasis to the lungs of tumor‐bearing mice. Mice were injected in the fourth mammary fat pad or the right back with tdTomato‐labeled E0771, tdTomato‐labeled LLC or EGFP‐labeled B16F10 cells, respectively, and the lungs were removed after perfusion at 7, 10, 14, 18, or 22 days. (B–D) Analysis of spontaneous metastasis for tdTomato‐labeled E0771 (B), tdTomato‐labeled LLC (C), or EGFP‐labeled B16F10 cells (D). Representative flow cytometric analysis of lung cells is shown, with cells corresponding to metastases (tdTomato^+^ or EGFP^+^ cells) being outlined by black boxes. (E) Summary of results for the timing of spontaneous metastasis. Day 5 after LLC or B16F10 cell transplantation or day 7 after E0771 cell transplantation is defined as the early premetastatic phase, day 7 after LLC or B16F10 cell transplantation or day 14 after E0771 cell transplantation as the late premetastatic phase, and day 14 after LLC or B16F10 cell transplantation or day 21 after E0771 cell transplantation as the micrometastatic phase.

**FIGURE 2:**
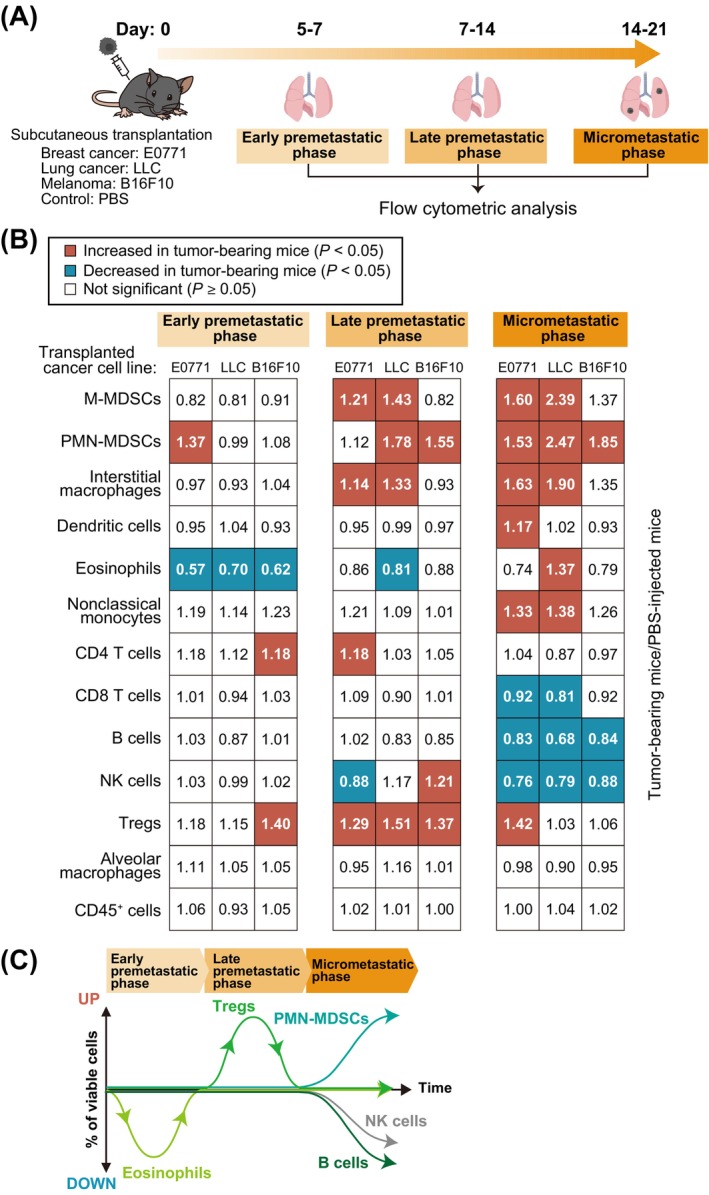
Generality of changes in lung immune cell composition associated with metastasis. (A) Schematic representation for flow cytometric analysis of lung immune cells from tumor‐bearing mice. Mice were injected in the fourth mammary fat pad or the right back with E0771, LLC or B16F10 cells or with phosphate‐buffered saline (PBS) as a control, and lung cells were isolated for analysis after 5–21 days. (B) Summary of changes in lung immune cell composition during the early premetastatic, late premetastatic, and micrometastatic phases. Significant (*p* < 0.05) increases or decreases relative to PBS‐injected mice are indicated by red and blue squares, respectively (unpaired two‐sided Student's *t* test or Tukey–Kramer test). The numbers in the squares indicate the ratio of the percentage of each cell type among viable cells for tumor‐bearing mice to that for PBS‐injected mice and are means from 6 to 41 mice. (C) Summary of the dynamics of lung immune cell types showing a decrease in the number of eosinophils in the early premetastatic phase, an increase in Tregs in the late premetastatic phase, and an increase in PMN‐MDSCs and a decrease in B cells and NK cells in the micrometastatic phase.

To examine the pattern of changes in immune cell types in the lungs associated with metastasis, we first developed experimental mouse models for the study of premetastatic to early metastatic phases. We injected mice subcutaneously with tdTomato‐labeled E0771 breast cancer cells, tdTomato‐labeled LLC lung cancer cells, or EGFP‐labeled B16F10 melanoma cells, subsequently collected lung cells, and checked for spontaneous metastasis (tdTomato^+^ or EGFP^+^ cells) by flow cytometric analysis (Figure 1A–D). Based on these results, we defined three time points (early premetastatic, late premetastatic, and micrometastatic phases) (Figure 1E). Spontaneous metastasis to the lungs was not observed for any of the injected mice in the early premetastatic phase or in the late pre‐metastatic phase, except for one mouse, whereas it was apparent for all mice in the micrometastatic phase. Comprehensive flow cytometric analysis of lung immune cells revealed distinctive profiles of cell composition at each metastatic phase that were common among E0771, LLC and B16F10 models (Figure 2A,B and Figures S1 and S2). The proportion of eosinophils was decreased in the early premetastatic phase, that of Tregs was increased in the late premetastatic phase, and that of PMN‐MDSCs was increased and that of B cells and natural killer (NK) cells was each decreased in the micrometastatic phase (Figure 2C). Although IL‐5 is a cytokine that specifically induces differentiation of eosinophils [21], an enzyme‐linked immunosorbent assay (ELISA) showed no change in serum IL‐5 levels in the early premetastatic phase (Figure S3), suggesting that the decline in eosinophil numbers during this phase is independent of IL‐5.

**FIGURE 3:**
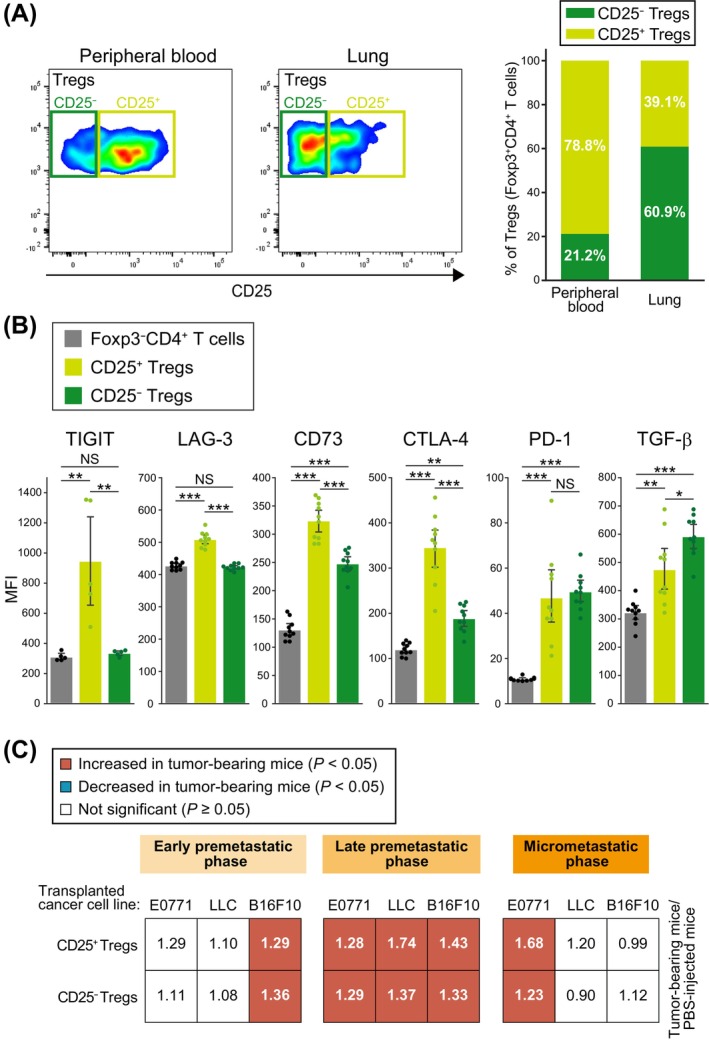
Both CD25^−^ Tregs and CD25^+^ Tregs accumulate in the lungs during the late premetastatic phase. (A) Representative flow cytometric analysis of CD25 expression in Tregs (Foxp3^+^CD4^+^ T cells) of peripheral blood or lung of control mice (left). Percentage of CD25^+^ or CD25^−^ cells among Tregs (right). Data are means from 5 mice. (B) Flow cytometric quantification of TIGIT, LAG‐3, CD73, CTLA‐4, PD‐1, and TGF‐β expression in Tregs and Foxp3^−^CD4^+^ T cells isolated from the lung of control mice. MFI, mean fluorescence intensity. Data are means +95% confidence interval (*n* = 5 to 10 mice). *p < 0.05, **p < 0.01, ***p < 0.001; NS, not significant (Tukey–Kramer test). (C) Summary of changes in the numbers of CD25^+^ Tregs and CD25^−^ Tregs in the lung during premetastatic to early metastatic phases. Significant (*p* < 0.05) increases or decreases relative to PBS‐injected mice are indicated by red and blue squares, respectively (unpaired two‐sided Student's *t* test or Tukey–Kramer test). The numbers in the squares indicate the ratio of the percentage of each cell type among viable cells for mice injected with cancer cell lines to that for PBS‐injected mice and were derived from 8 to 34 mice.

Tregs are classified into two separate cell types according to whether they express CD25 or not, with CD25^−^ Tregs being predominant in the lung [22]. We found that CD25^+^ Tregs and CD25^−^ Tregs were predominant in peripheral blood and the lungs, respectively, of control mice (Figure 3A), and that CD25^−^ Tregs in the lungs expressed lower levels of immune checkpoint molecules (TIGIT, LAG‐3, and CTLA‐4) but higher levels of TGF‐β compared with corresponding CD25^+^ Tregs (Figure 3B). We also confirmed that the numbers of both CD25^−^ Tregs and CD25^+^ Tregs in the lungs were increased in the late premetastatic phase (Figure 3C). These results suggested that CD25^−^ Tregs and CD25^+^ Tregs might contribute to the formation of the premetastatic niche by different mechanisms. To elucidate the mechanism responsible for the increase in Tregs in the late premetastatic phase, we performed ELISAs for the chemokines CCL17, CCL22, and CCL2. CCL17 and CCL22 promote the homing of Tregs to the lungs [23], whereas CCL2 is required for the recruitment of Tregs to tumor sites [24]. No common changes in the levels of CCL17 and CCL22 were apparent in the lung or peripheral blood, with the concentration of CCL17 being increased only in the peripheral blood of B16F10 cell‐transplanted mice (Figure S4A). In contrast, an increase in CCL2 levels was observed in lung and peripheral blood of LLC cell‐transplanted mice and in peripheral blood of E0771 cell‐transplanted mice. We confirmed by flow cytometric analysis that ~10% of both CD25^−^ Tregs and CD25^+^ Tregs in both peripheral blood and lung of control mice expressed the CCL2 receptor CCR2 (Figure S4B). However, an increase in the proportion of Tregs in the late premetastatic phase was also observed in the lungs of mice deficient in CCR2 (Figure S4C–E). Collectively, these findings thus suggested that the accumulation of Tregs in lung during the late premetastatic phase is independent of CCL17, CCL22, and CCL2.

### 3.2 Changes in Immune Cell Composition in Lung Are Similar to Those in Peripheral Blood

**FIGURE 4:**
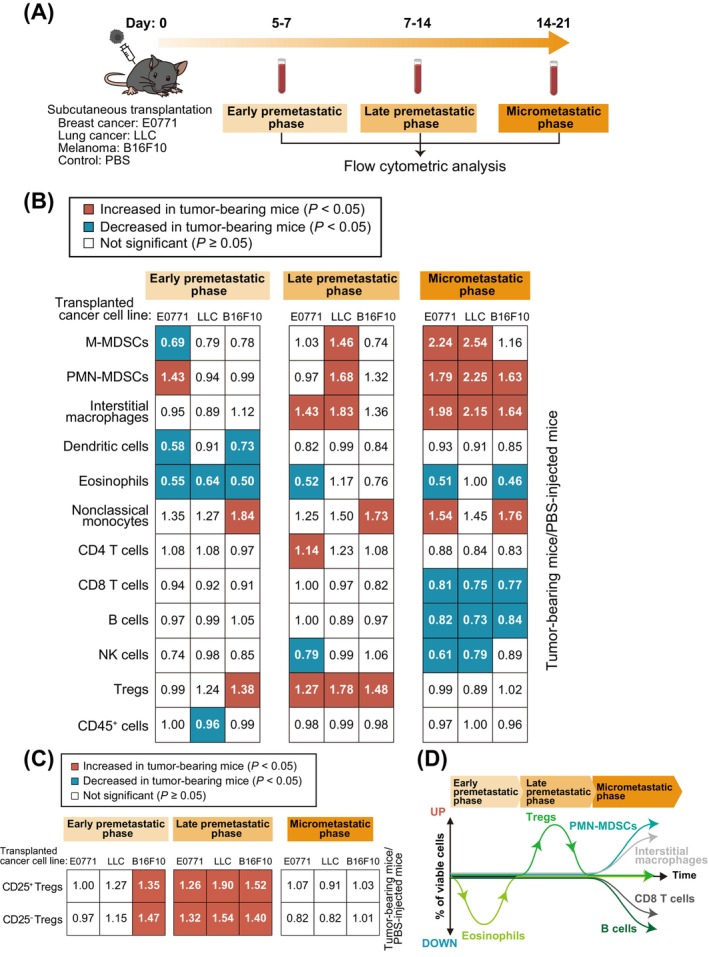
Changes in immune cell composition associated with metastasis for peripheral blood are similar to those for lung. (A) Schematic representation for flow cytometric analysis of immune cells from peripheral blood of tumor‐bearing mice. Mice were injected in the fourth mammary fat pad or the right back with E0771, LLC or B16F10 cells or with PBS (control), and peripheral blood was collected from the right ventricle of mice before perfusion at 5–21 days. (B) Summary of changes in immune cell composition for peripheral blood during premetastatic to early metastatic phases. Significant (*p* < 0.05) increases or decreases relative to PBS‐injected mice are indicated by red and blue squares, respectively (unpaired two‐sided Student's *t* test or Tukey–Kramer test). The numbers in the squares indicate the ratio of the percentage of each cell type among viable cells for tumor‐bearing mice to that for PBS‐injected mice (*n* = 4 to 29 mice). (C) Summary of changes in the numbers of CD25^−^ Tregs and CD25^+^ Tregs in peripheral blood from premetastatic to early metastatic phases. Significant (*p* < 0.05) increases relative to PBS‐injected mice are indicated by red squares (unpaired two‐sided Student's *t* test or Tukey–Kramer test). The numbers in the squares indicate the ratio of the percentage of each cell type among viable cells in tumor‐bearing mice to that in PBS‐injected mice (*n* = 6 to 20 mice). (D) Summary of the dynamics of immune cell types in peripheral blood showing a decrease in the number of eosinophils in the early premetastatic phase, an increase in Tregs in the late premetastatic phase, and an increase in PMN‐MDSCs and interstitial macrophages and a decrease in B cells and CD8 T cells in the micrometastatic phase.

We next performed comprehensive flow cytometric analysis of immune cells in the peripheral blood of tumor‐bearing mice (Figure 4A). We found that the proportion of eosinophils was decreased in the early premetastatic phase, that of Tregs was increased in the late premetastatic phase, and that of PMN‐MDSCs or interstitial macrophages was increased and that of B cells or CD8 T cells was decreased in the micrometastatic phase (Figure 4B,D and Figure S5). The proportions of both CD25^−^ Tregs and CD25^+^ Tregs were also increased in the late premetastatic phase (Figure 4C).

**FIGURE 5:**
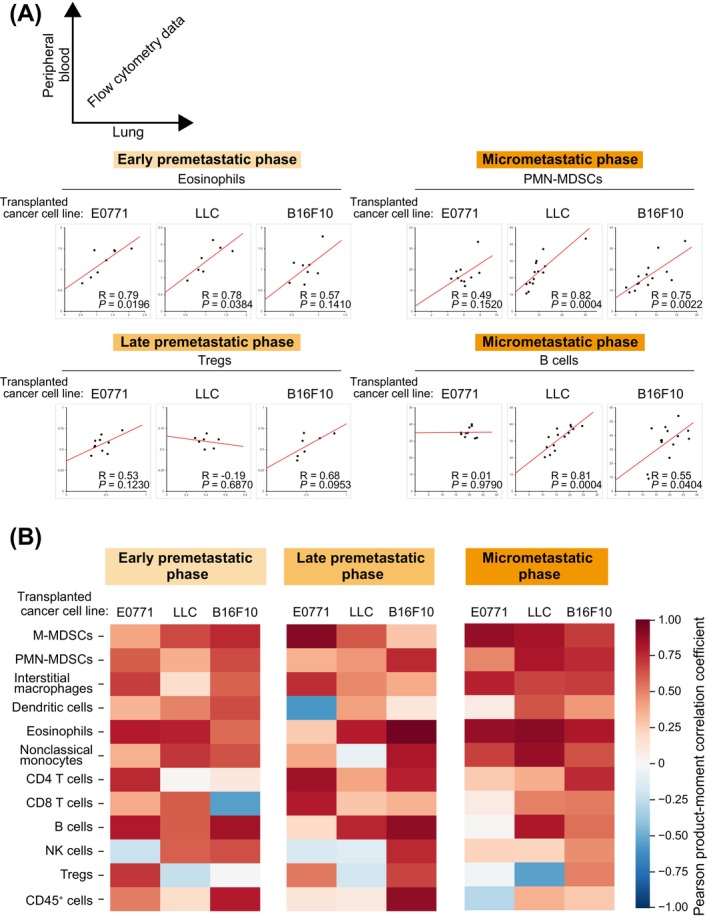
Correlation of immune cell changes in peripheral blood with those in the lung for the mouse models of metastasis. (A) Scatter plots for the percentage of the indicated immune cell types in peripheral blood (vertical axis) versus that in lung (horizontal axis) for the indicated premetastatic to early metastatic phases. Red lines represent the approximate straight lines. The correlation coefficient (*R*) is shown (*n* = 7 to 14 mice). (B) Heat map of Pearson product–moment correlation coefficients for lung immune cells versus peripheral blood immune cells (*n* = 7 to 14 mice).

Given the similar patterns of the changes in immune cell composition in both lung and peripheral blood, we calculated correlation coefficients for these changes. In most instances, the numbers of eosinophils, Tregs, PMN‐MDSCs, or B cells showed a significant positive correlation or tended to show such a correlation between lung and peripheral blood (Figure 5A). We also calculated correlation coefficients for all immune cells and found that the percentages of most cell types in the peripheral blood at the various phases were positively correlated with those in the lung (Figure 5B). These results suggested that the numbers of immune cells in the lung from premetastatic to early metastatic phases were determined by the numbers of these cells circulating in blood, and that the numbers of lung immune cells can be predicted from the numbers of peripheral blood immune cells.

### 3.3 Metastatic Phase Can be Predicted From Immune Cell Profiles

**FIGURE 6:**
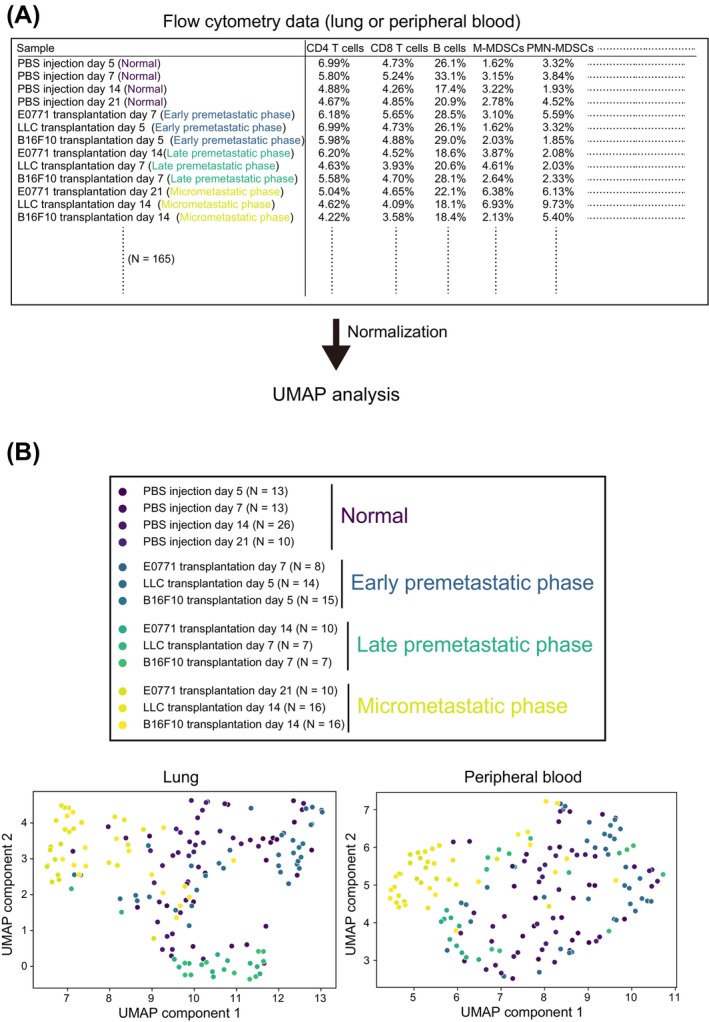
Each metastatic phase has a distinct immune cell profile. (A) Schematic diagram for UMAP analysis based on flow cytometry data. Normal phase corresponds to mice injected with PBS instead of cancer cells. (B) UMAP analysis based on normalized flow cytometry data.

**FIGURE 7:**
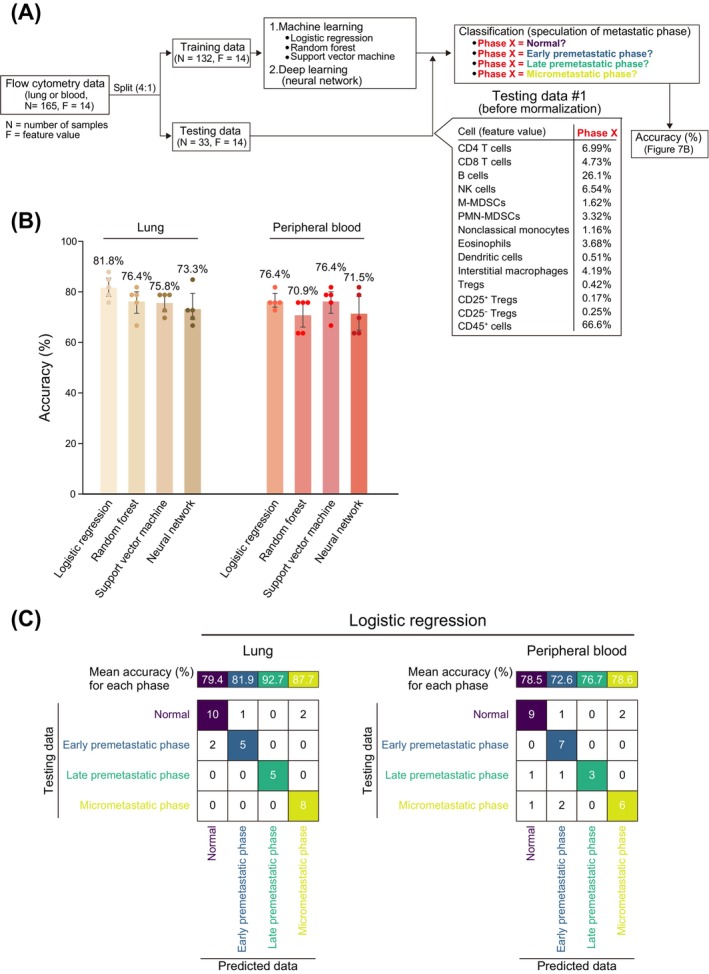
Prediction of metastatic phase from comprehensive flow cytometric data for immune cells. (A) Workflow for prediction of metastatic phase with the use of machine learning or deep learning. Normalized flow cytometry data (lung or peripheral blood) was split into training data or testing data at a ratio of 4:1, and training data were applied to machine learning or deep learning. The testing data were then used to predict metastatic phase and to calculate the prediction accuracy. The normalized flow cytometry data were randomly split five times, with learning and testing (prediction) by machine learning or deep learning also being performed five times in a corresponding manner and the mean accuracy calculated. (B) Accuracy of metastatic phase prediction. Data are means +95% confidence interval (*n* = 5), with the mean accuracy also being shown above each bar. (C) Mean accuracy for prediction of each metastatic phase by logistic regression and an example of the prediction results.

We performed a uniform manifold approximation and projection (UMAP) analysis to observe differences in immune cell profiles for the various metastatic phases (Figure 6A). Each metastatic phase was found to have a distinct immune cell profile, with these differences being especially prominent in the lung (Figure 6B). We therefore examined whether flow cytometric data for immune cells are able to predict metastatic phase with the use of machine learning (logistic regression, random forest, or support vector machine) or deep learning (neural network) (Figure 7A). All methods showed an accuracy of ~75%, with logistic regression having the highest accuracy of 81.8% for lung and 76.4% for peripheral blood (Figure 7B). The accuracy for the different phases was highest for late premetastatic and micrometastatic phases in the lung (92.7% and 87.7%, respectively) (Figure 7C), consistent with the UMAP results (Figure 6B).

## 4 Discussion

The premetastatic niche has been considered a promising target for prevention of cancer metastasis. However, temporal information regarding which cell types accumulate in what order in the premetastatic niche has been lacking, and the generality of such accumulation patterns has remained unclear. Organ thin sections and HE staining are typical methods for evaluating cancer metastasis. However, it is impractical to prepare thin sections of the entire organ and examine all sections using HE staining to detect a small number of tumor cells. To address this limitation, we adopted flow cytometric analysis to detect micrometastases at the few cell level. We identified common changes in immune cell profiles (decrease in eosinophils in the early premetastatic phase, increase in Tregs in the late premetastatic phase, and increase in PMN‐MDSCs and decrease in B cells in the micrometastatic phase) that were independent of cancer type.

Although a generalized decrease in neutrophils, eosinophils, and basophils (granulocytopenia) is common in patients with infectious diseases, autoimmune diseases, and those undergoing drug or radiation therapy [25], a specific decrease in eosinophils (eosinopenia) is uncommon. Eosinopenia is known to be seen in typhoid fever [26], but the mechanism is not understood. In the present study, we focused on the cytokine IL‐5, which is known to specifically increase eosinophils but found no changes. The possibility of medication, cortisol or stress levels, inflammatory responses, or secondary effects of the experiment cannot be ruled out, but it is unlikely that these possibilities would cause only eosinophils to decrease specifically and not granulocytes in general. Cytokines such as GM‐GSF, IL‐3, and CCL11 might be involved, although they are less specific for eosinophils than IL‐5. Although M‐MDSCs and PMN‐MDSCs are well‐characterized components of the premetastatic niche [27], we found that the numbers of these cells were not commonly increased in the late premetastatic phase of mice transplanted with the three types of cancer cells. M‐MDSCs and PMN‐MDSCs also promote Treg differentiation [7]. but our results suggested that Tregs accumulated in the lung before each of these cell types. Tumor cell‐derived TGF‐β was shown to promote MDCSs differentiation and proliferation [27]. We confirmed that Tregs produce TGF‐β, and Tregs‐derived TGF‐β may contribute to the increase in MDSCs in the micrometastatic phase. The effect of the transient increase in Tregs apparent in the late premetastatic phase on cancer metastasis remains unclear and warrants further study. Our results may provide a basis for the development of more generalized methods to prevent cancer metastasis that are applicable to all solid tumors.

Our finding that changes in the immune cell composition of peripheral blood were similar to those apparent for the lungs suggested that these changes in peripheral blood and lung may be linked and that it might be possible to predict lung metastatic phase from comprehensive flow cytometric data for peripheral blood immune cells with the use of machine learning or deep learning approaches. However, the mean accuracy of such prediction was ~75%, which might not be high enough for clinical application. Given that we used only 165 samples for machine learning and deep learning in this study, however, it may be possible to improve the accuracy with the use of a larger number of samples. Moreover, we used immune cell data from specific time points to make predictions, but long‐term monitoring of peripheral immune cells and the addition of time information might allow more accurate predictions. To validate our findings in humans, we need data on the presence or absence of micrometastases in patients and comprehensive and temporal information on immune cells, but we were unable to find a public dataset that meets these two requirements. A previous study attempted to detect hepatocellular carcinoma or pancreatic ductal adenocarcinoma by machine learning (random forest) with data obtained by mass cytometric time‐of‐flight (CyTOF) analysis of peripheral blood immune cells from 2348 individuals, including 790 and 376 patients with these cancer types, respectively, and 633 healthy volunteers [28]. The presence or absence of a tumor could be predicted with a sensitivity of ~80%. The combination of the CYTOF data with circulating levels of α‐fetoprotein or carbohydrate antigen 19–9 (CA19‐9), both known tumor markers, resulted in an increased sensitivity of up to 88%. These results suggest that immune cell profiles of peripheral blood may be useful in predicting cancer metastasis in humans. Previous studies also suggest that data on blood proteins, extracellular vesicles, DNA, RNA, or metabolites may be useful for more accurate prediction.

In summary, there is a generality of changes in immune cell profiles from premetastatic to early metastatic phases in the lung, which may allow prediction of metastatic phase and provide a basis for the development of methods for early detection and prevention of cancer metastasis in a manner independent of cancer type.

## Author Contributions

**Shigeaki Sugiyama:** conceptualization (lead), data curation (lead), formal analysis (lead), methodology (lead), resources (lead), software (lead), validation (lead), visualization (lead), writing – original draft (lead), writing – review and editing (supporting). **Kanae Yumimoto:** conceptualization (supporting), project administration (supporting), supervision (supporting), writing – original draft (supporting), writing – review and editing (supporting). **Keiichi I. Nakayama:** conceptualization (supporting), funding acquisition (lead), project administration (lead), supervision (lead), writing – original draft (supporting), writing – review and editing (lead).

## Ethics Statement

All animal experiments were approved by the animal ethics committee of Kyushu University (approval codes A22‐242‐0 and A22‐242‐1) and were conducted in compliance with university guidelines and regulations for animal experimentation.

## Conflicts of Interest

The authors declare no conflicts of interest.

## Supporting information


**Figure S1.** Gating strategies for immune cell analysis.
**Figure S2.** Comprehensive flow cytometric analysis of lung immune cells from mice injected with cancer cell lines or PBS.
**Figure S3.** Serum IL‐5 levels in the early premetastatic phase.
**Figure S4.** The accumulation of Tregs in the lungs during the late premetastatic phase is independent of CCL17, CCL22, and CCL2.
**Figure S5.** Comprehensive flow cytometric analysis of immune cell types in peripheral blood of mice injected with cancer cell lines or PBS.

## Data Availability

All data generated or analyzed during this study are included in this article. Further inquiries can be directed to the corresponding author.
