# Supplementary material for: Immune Cell Profiling Reveals a Common Pattern in Premetastatic Niche Formation Across Various Cancer Types
Source: Cancer Med. 2024 Dec 30;14(1):e70557. doi: 10.1002/cam4.70557 (PMC11683674; doi:10.1002/cam4.70557)
Supplement: Supplementary file 1 — Figure S1. Gating strategies for immune cell analysis. Figure S2. Comprehensive flow cytometric analysis of lung immune cells from mice injected with cancer cell lines or PBS. Figure S3. Serum IL‐5 levels in the early premetastatic phase. Figure S4. The accumulation of Tregs in the lungs during the late premetastatic phase is independent of CCL17, CCL22, and CCL2. Figure S5. Comprehensive flow cytometric analysis of immune cell types in peripheral blood of mice injected with cancer cell lines or PBS. [file CAM4-14-e70557-s001.pdf]

Supplementary Figures

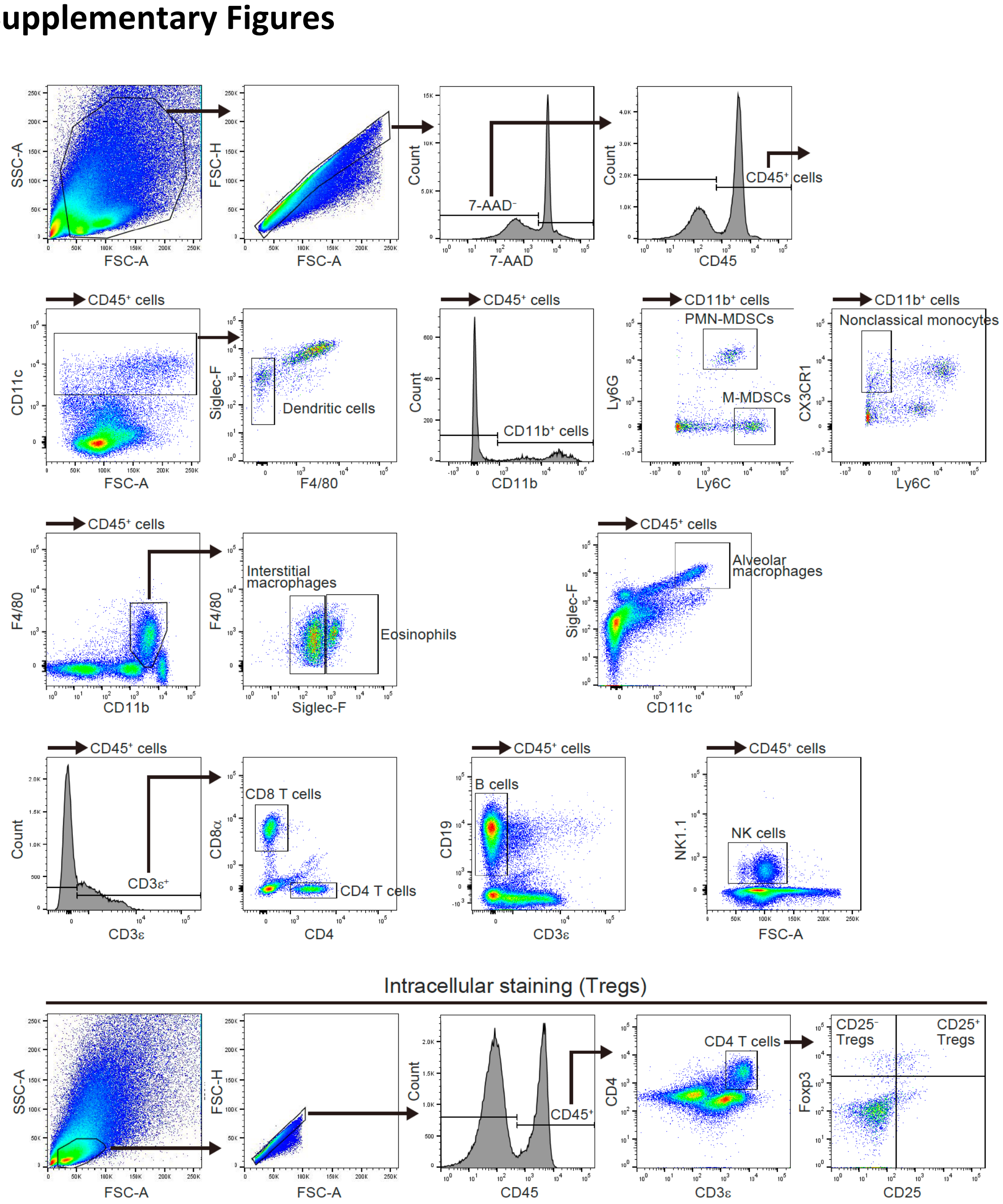

Figure S1

Gating strategies for immune cell analysis. Debris was first removed on the basis of forward scatter (FSC-A) and side scatter (SSC-A), single cells were then gated on the basis of FSC-A and FSC-H, and viable cells were gated on the basis of 7-AAD staining (with this process being skipped for intracellular staining of Foxp3). Immune cells were further gated on the basis of CD45 expression. The indicated immune cell types were gated on the basis of specific surface markers as shown. The proportion of Tregs (% of viable cells) was obtained from the product of Tregs (% of CD4 T cells) and CD4 T cells (% of viable cells).

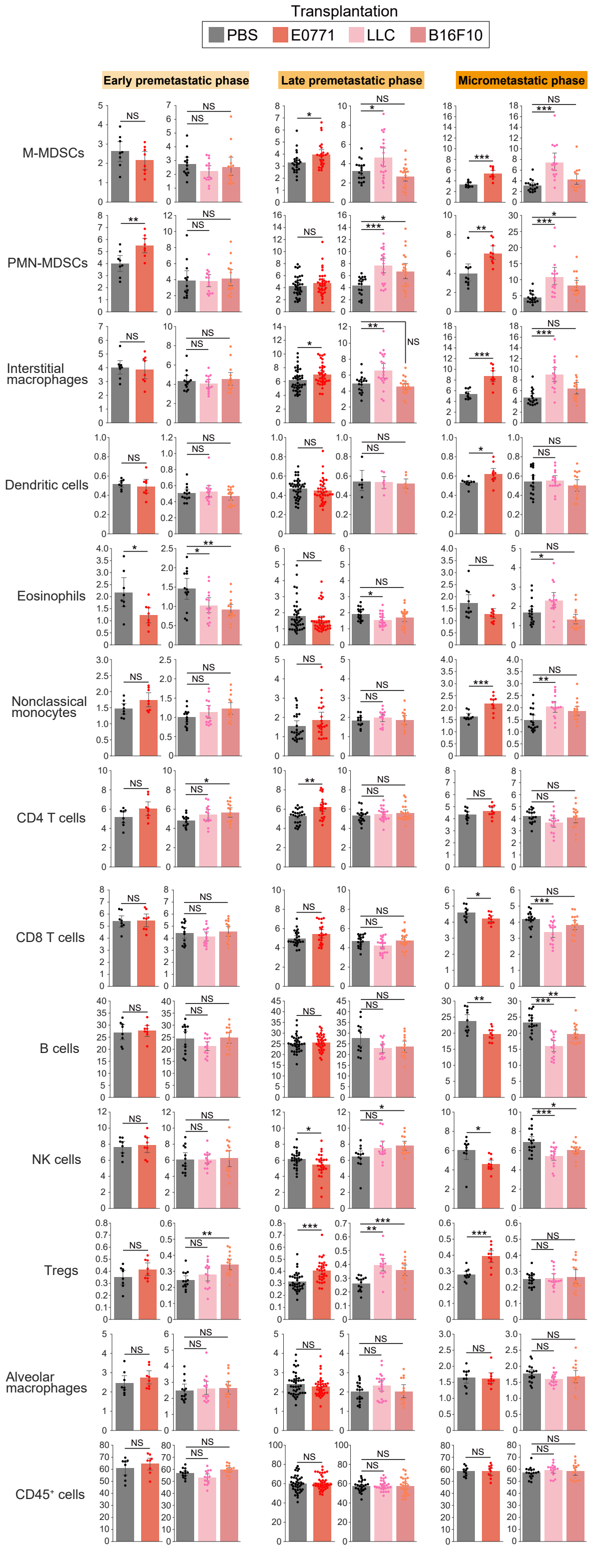

Transplantation
PBS
E0771
LLC
B16F10
Early premetastatic phase
Late premetastatic phase
Micrometastatic phase
M-MDSCs
PMN-MDSCs
Interstitial macrophages
Dendritic cells
Eosinophils
Nonclassical monocytes
CD4 T cells
CD8 T cells
B cells
NK cells
Tregs
Alveolar macrophages
CD45⁺ cells

Figure S2

Comprehensive flow cytometric analysis of lung immune cells from mice injected with cancer cell lines or PBS. A summary of these data is shown in Figure 2B. The vertical axis shows the percentage of each immune cell type among viable cells. Data are means + 95% confidence interval (n = 6 to 41 mice). NS, not significant. *$P < 0.05$, **$P < 0.01$, ***$P < 0.001$ (unpaired two-sided Student's t test or Tukey-Kramer test).

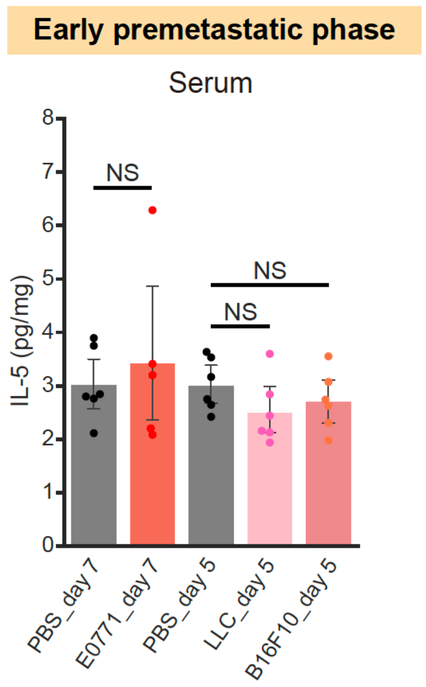

Figure S3

Serum IL-5 levels in the early premetastatic phase. Serum IL-5 was measured with an ELISA at the indicated times after tumor cell or PBS injection. Data are means + 95% confidence interval (*n* = 5 or 6 mice). NS, not significant (unpaired two-sided Student's t test or Tukey-Kramer test).

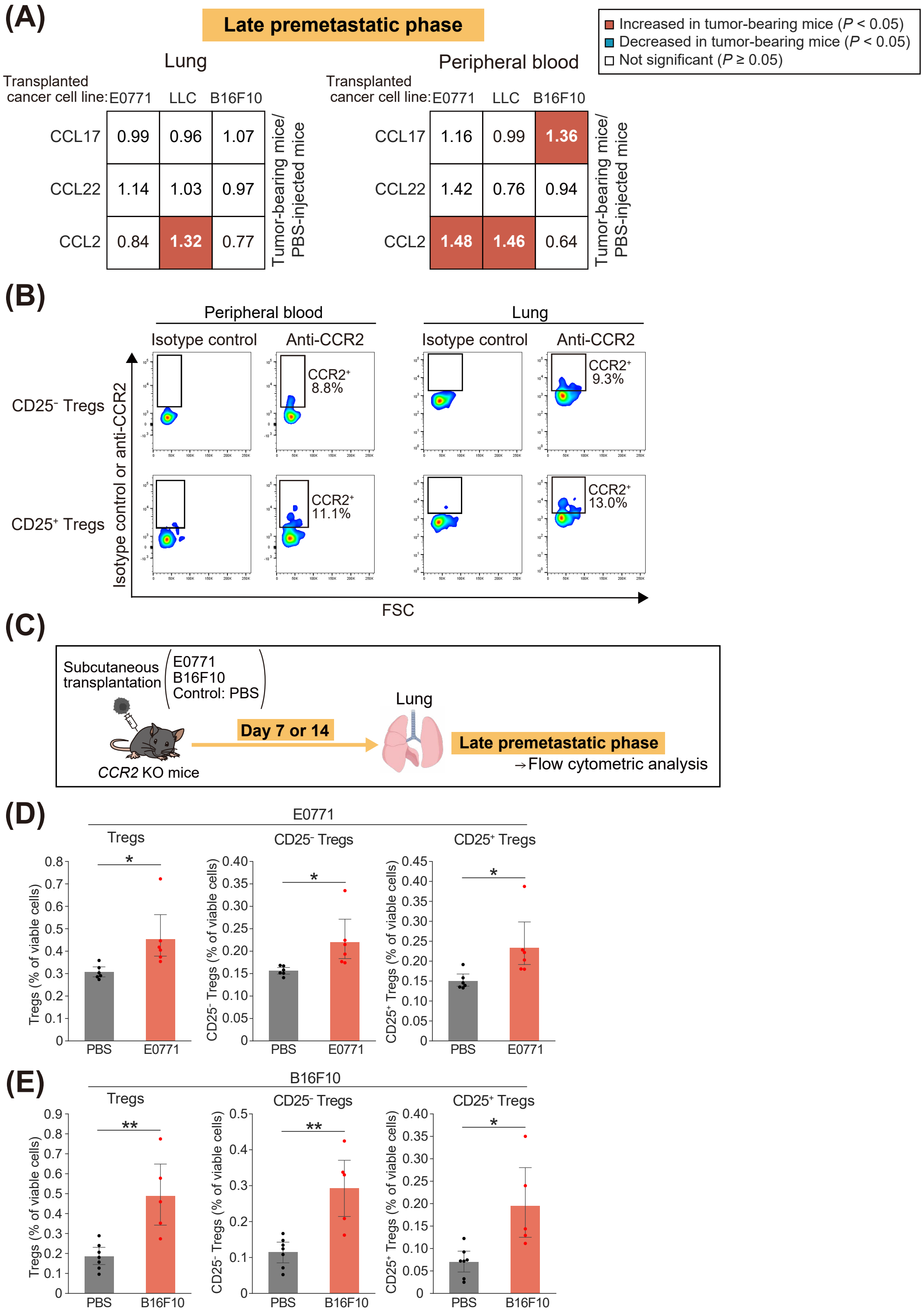

(A)
Late premetastatic phase
Increased in tumor-bearing mice (P < 0.05)
Decreased in tumor-bearing mice (P < 0.05)
Not significant (P ≥ 0.05)
Lung
Transplanted cancer cell line: E0771 LLC B16F10
CCL17 0.99 0.96 1.07
CCL22 1.14 1.03 0.97
CCL2 0.84 1.32 0.77
Tumor-bearing mice/ PBS-injected mice
Peripheral blood
Transplanted cancer cell line: E0771 LLC B16F10
CCL17 1.16 0.99 1.36
CCL22 1.42 0.76 0.94
CCL2 1.48 1.46 0.64
Tumor-bearing mice/ PBS-injected mice
(B)
Peripheral blood
Lung
Isotype control
Anti-CCR2
CD25⁻ Tregs
CD25⁺ Tregs
CCR2⁺ 8.8%
CCR2⁺ 9.3%
CCR2⁺ 11.1%
CCR2⁺ 13.0%
Isotype control or anti-CCR2
FSC
(C)
Subcutaneous transplantation
E0771
B16F10
Control: PBS
CCR2 KO mice
Day 7 or 14
Lung
Late premetastatic phase
→Flow cytometric analysis
(D)
E0771
Tregs
CD25⁻ Tregs
CD25⁺ Tregs
Tregs (% of viable cells)
CD25⁻ Tregs (% of viable cells)
CD25⁺ Tregs (% of viable cells)
PBS
E0771
*
(E)
B16F10
Tregs
CD25⁻ Tregs
CD25⁺ Tregs
Tregs (% of viable cells)
CD25⁻ Tregs (% of viable cells)
CD25⁺ Tregs (% of viable cells)
PBS
B16F10
**
*

Figure S4

The accumulation of Tregs in the lungs during the late premetastatic phase is independent of CCL17, CCL22, and CCL2. (A) Summary of changes in the levels of CCL17, CCL22, and CCL2 as measured by ELISAs in the lungs and peripheral blood during the late premetastatic phase. Significant ($P < 0.05$) increases or decreases relative to PBS-injected mice are indicated by red and blue squares, respectively (unpaired two-sided Student's *t* test or Tukey-Kramer test). The numbers in the squares indicate the ratio of the concentration of each protein (w/w) in tumor-bearing mice to that in PBS-injected mice (n = 5 or 6 mice). (B) Representative flow cytometric analysis of CCR2 expression in $CD25^{+}$ Tregs and $CD25^{-}$ Tregs of peripheral blood or lung of control mice. (C) Schematic representation for flow cytometric analysis of lung Tregs from tumor-bearing *CCR2* knockout (KO) mice. The mice were injected in the fourth mammary fat pad or the right back with E0771 or B16F10 cells or with PBS (control), and lung cells were isolated after perfusion at 7 (B16F10) or 14 (E0771) days. (D and E) Flow cytometric quantification of lung Tregs from *CCR2* KO mice injected with E0771 (D) or B16F10 (E) cells. Quantitative data are means + 95% confidence interval (n = 6 mice in D; n = 5 (B16F10) or 7 (PBS) mice in E). *$P < 0.05$, **$P < 0.01$ (unpaired two-sided Student's *t* test).

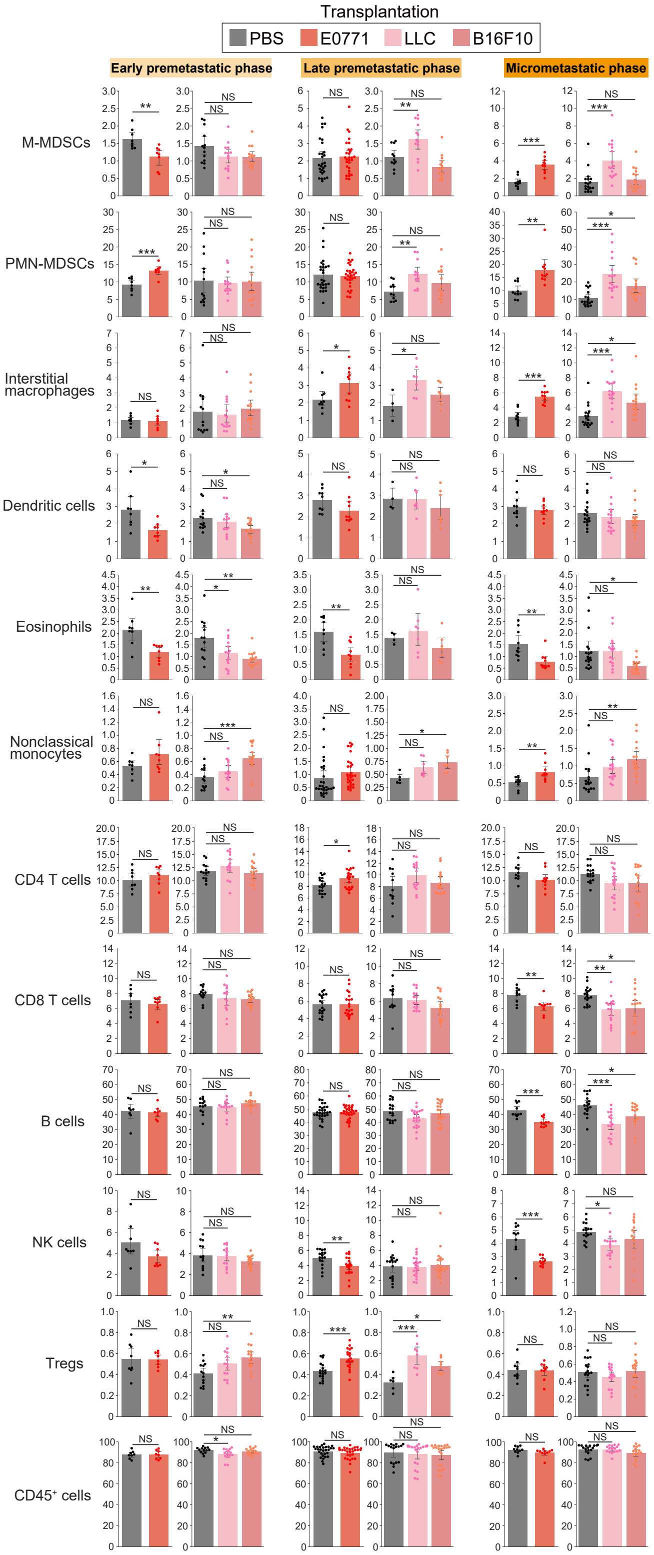

Transplantation
PBS
E0771
LLC
B16F10
Early premetastatic phase
Late premetastatic phase
Micrometastatic phase
M-MDSCs
PMN-MDSCs
Interstitial macrophages
Dendritic cells
Eosinophils
Nonclassical monocytes
CD4 T cells
CD8 T cells
B cells
NK cells
Tregs
CD45⁺ cells

Figure S5

Comprehensive flow cytometric analysis of immune cell types in peripheral blood of mice injected with cancer cell lines or PBS. These data are summarized in Figure 4B. The vertical axis shows the percentage of each immune cell type among viable cells. Data are means + 95% confidence interval (n = 4 to 29 mice). *$P < 0.05$, **$P < 0.01$, ***$P < 0.001$; NS, not significant (unpaired two-sided Student's t test or Tukey-Kramer test).
